# Predictors of Performance on the United States Medical Licensing Examination Step 2 Clinical Knowledge: A Systematic Literature Review

**DOI:** 10.7759/cureus.22280

**Published:** 2022-02-16

**Authors:** Adrian Jacobparayil, Hisham Ali, Brian Pomeroy, Regina Baronia, Marina Chavez, Yasin Ibrahim

**Affiliations:** 1 Psychiatry, Texas Tech University Health Sciences Center, Lubbock, USA; 2 Pediatric Medicine, Texas Tech University Health Sciences Center, Lubbock, USA

**Keywords:** usmle step 2 ck, performance predictors, medical student, test preparation, usmle step 1 pass/fail

## Abstract

In February 2020, the governing bodies of the United States Medical Licensing Examination (USMLE) announced the decision to change Step 1 score reporting from a three-digit system to pass/fail designation. Previous studies theorized that Step 2 Clinical Knowledge (CK) will become the numerical standard by which residency directors can quickly sort through program applicants. The goal of this study is to review prior research and identify significant factors associated with Step 2 CK outcomes.

A systematic literature search on PubMed, Web of Science, Scopus, and ERIC that included articles published between 2005 and 2015 was conducted using the keywords “USMLE,” “Step 2 CK,” “score,” “success,” and “predictors.”

After screening the initial search yield of 3,239 articles, 52 articles were included for this review. Positively correlated factors included Step 1 score, clinical block grades, Comprehensive Clinical Science Self-Assessment (CCSSA), Comprehensive Clinical Science Examination (CCSE), and volunteerism. Factors such as clerkship sequence and pass/fail grading failed to correlate with Step 2 CK. Medical College Admission Test (MCAT) score (p < 0.01) and undergraduate grade point average (GPA) (p = 0.01) positively correlated, while age displayed a negative correlation. Additionally, females typically scored higher on Step 2 CK than their male peers.

The study findings suggest that continuous learning and academic success throughout medical school positively influence eventual Step 2 CK scoring. Performance on USMLE practice examinations, Step 1, and clinical evaluations serve as positive predictors for Step 2 CK scores. Interestingly, changing answers and spending more time on each question during the examination were associated with higher scores.

## Introduction and background

The United States Medical Licensing Examination (USMLE) consists of a series of required examinations for medical practice in the United States. Step 1 assesses a student’s ability to apply basic science principles, and Step 2 Clinical Knowledge (CK) assesses the ability to apply medical knowledge to patient care situations in a clinical setting [1]. Passing grades for both examinations are required for medical school graduation and progression into residency [1,2].

In February 2020, the governing bodies of the USMLE, the Federation of State Medical Board (FSMB), and the National Board of Medical Examiners (NBME) announced the decision to change Step 1 score reporting from a three-digit system (1-300) to a pass/fail designation [1]. This change was designated to be implemented on January 1, 2022. With the loss of one objective method for residency distinction, it may occur that Step 2 CK will become a more important metric that residency directors will use to quickly evaluate program applicants.

It is important to identify and examine factors that have an impact on the medical students’ performance in the Step 2 CK examination for both medical institutions and students. The goal of this study is to review prior research and identify significant factors positively and negatively associated with Step 2 CK outcomes. Our findings can help medical students, instructors, and medical schools ascertain the variables most likely to ensure successful performance on Step 2 CK.

This article was previously presented as a poster presentation at the 2021 APA 2021 Annual Meeting on May 2, 2021.

Methods

**Figure 1:**
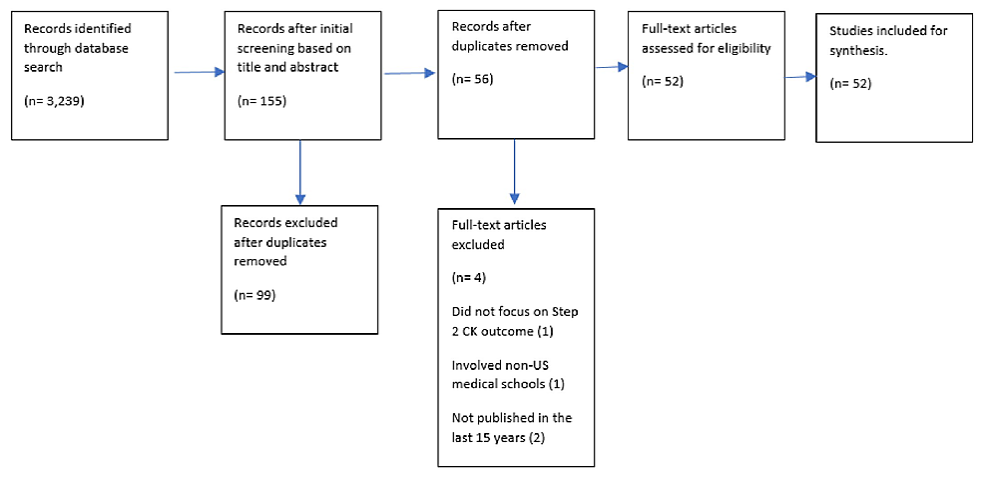
PRISMA flowchart

A systematic literature search was conducted using PubMed, Web of Science, Scopus, and ERIC. The keywords were a combination of the following: USMLE, Step-2 CK, score, success, and predictors. Our criteria included articles published within the last 15 years (2005-2020), with the most recent publication in January 2020. Additional criteria specified that selected articles must focus on USMLE Step 2 CK outcomes and include allopathic medical schools located in the United States. Duplicates and nonscientific papers were also removed (Figure 1). Each publication was reviewed independently and summarized in a separate excel table that was later synthesized to the abovementioned PRISMA flowchart. We compared findings to filter out articles based on the exclusion criteria and resolve inconsistencies. We discuss the impact of biases after analyzing the final 52 articles and took them into consideration when evaluating the relationships between variables. Variables from the articles were categorized into either modifiable or unmodifiable factors.

Variables that can be altered during attendance in medical school until the first Step 2 CK attempt were considered as modifiable factors. These were further divided into individual and institutional modifiable variables. Individual factors include stress, Step 1 score, and clinical block grades. Institutional variables include clerkship sequence, faculty-to-student ratio, and pass/fail grading. Unmodifiable variables occur prior to enrollment into medical school and cannot be modified before the first Step 2 CK attempt. These variables include age, gender, race, and Medical College Admission Test (MCAT) score.

## Review

Results

The initial literature search yielded 3,239 articles, which were then narrowed down to 155 articles after reading the title and abstract. All articles that did not meet the criteria outlined in the Methods section were excluded. This exclusion resulted in 52 articles that were reviewed and agreed upon by all authors of this study. Most of the included articles (47) focused on modifiable factors as compared to unmodifiable factors (23). There was some overlap between the above articles.

Many of the included studies examined variables such as MCAT score, undergraduate grade point average (GPA), Step 1 score, and clinical grades. These variables were similar in that they all were strongly correlated to Step 2 CK scoring. There were also several unique variables that will be highlighted below. The above common variables, in addition to demographic variables, were controlled in many of these studies. At one California medical school, students with objective socioeconomic disadvantage (SED) and subjective self-designated disadvantage (SDA) had lower mean Step 2 CK scores compared to their peers [3]. However, SED itself did not have a discernible effect on Step 2 CK scoring [3]. Pass/fail preclinical curriculum did not have a significant effect on Step 2 CK scoring [4,5]. Students participating in extracurricular activities, such as free clinic volunteering, were found to have higher Step 2 CK scores compared to their peers [6]. Students involved in peer-led tutoring had significantly different (p < 0.001) Step 2 CK scores, but confounding variables were not well controlled [7]. When preparing for the examination, one study found that a larger time gap between the end of clinical rotations and taking Step 2 CK (defined as lag time) was negatively correlated to examination scores [8]. When taking the examination, time per question [9] and changing answers [10] also had a positive correlation to the Step 2 CK score. Interestingly enough, another study found that self-reported stress during the third year did not correlate with Step 2 CK outcomes [11]. A detailed summary of results can be found in the Appendix (Table 1).

Discussion

This review has examined numerous research studies on multiple factors that have been correlated to performance in Step 2 CK. The objective of this review was to identify factors that could be addressed within the timeframe of students’ medical school education, and to this end, we have divided the reported factors into modifiable factors and unmodifiable factors. While there are many studies on these variables, we have purposely decided to keep this brief to focus on modifiable factors. These modifiable factors were further grouped into variables that can be addressed on the institutional level, e.g., curriculum, and individual variables, such as individual performance in practice examinations.

Modifiable Factors

Modifiable factors can be further separated into institutional and individual variables. Institutional variables involve factors that the medical school administration can change, such as faculty and curriculum characteristics. Individual variables involve factors that the medical student can alter or consider prior to their first Step 2 CK attempt.

Individual variables: These include USMLE practice examinations, USMLE Step 1, medical school assessments, Step 2 CK test day strategies, extracurricular activities, psychological variables, time spent in medical school, lag time, and study tools.

For USMLE practice examinations, several official practice examinations are available to students preparing for both Step 1 and Step 2 CK examinations. The Comprehensive Basic Science Examination (CBSE) is offered to students preparing for Step 1. CBSE score was positively correlated to Step 2 CK [12]. However, its use as a tool to gauge Step 2 CK competency would not have added value because it is a tool for Step 1 preparation.

The two practice examinations specific to Step 2 CK are the Comprehensive Clinical Science Examination (CCSE) and the Comprehensive Clinical Science Self-Assessment (CCSSA). The CCSSA, a self-assessment web-administered examination, is a good predictor for Step 2 CK scoring [13]. In particular, low CCSSA scores often indicated the danger of Step 2 CK failure [13]. The CCSE was also a significant positive predictor of Step 2 CK scoring [14,15]. In fact, a CCSE score of greater than 90 corresponded with a near 100% probability of passing Step 2 CK [14].

According to research, the predominant opinion is that the Step 1 score has a strong positive correlation with the Step 2 CK score [15,16]. There was also a significant correlation (r = 0.684, p ≤ 0.0001) between scoring higher than 208 on Step 1 and passing Step 2 CK on the first attempt [15]. In addition, for every 10-point increase in Step 1, a two-point increase in Step 2 CK was observed (p < 0.001) [12]. One study suggested that this positive relationship was stronger for males than for females [17]. The timing of the test is also an important variable to consider. One study found a unique relationship in that “students with lower MCAT scores performed better on Step 2 CK when Step 1 was after clerkships” [8]. However, in general, the researchers found that there was no significant relationship between the timing of the Step 1 test and Step 2 CK [8].

Medical school assessments included preclinical GPA, Objective Structured Clinical Examination (OSCE) evaluations, clinical block grades, and clinical NBME examination grades. Preclinical GPA had a small but positive impact on both Step 2 CK scoring and board certification [10,18]. Second- and third-year OSCE scores also had a weak correlation to Step 2 CK scoring [19]. In particular, scoring well in the differential diagnosis and identification of abnormalities skill subcomponents had a positive correlation with Step 2 CK scoring [20].

More impactful were the clinical block and NBME examination grades. Clinical block grades had a positive correlation (r = 0.517, p < 0.01) to Step 2 CK scoring [21]. Clinical NBME examination grades also had the same positive correlation (r = 0.77, p < 0.001) [22]. For example, every one-point increase on the surgical NBME led to an increase in the odds of passing Step 2 CK by 1.2 times [21]. In addition, failing and multiple attempts on the OB/GYN NBME were negatively correlated to Step 2 CK scoring (p = 0.008) [23].

Regarding Step 2 CK test day strategies, there seems to be a positive relationship with changing answers on Step 2 CK and score [24]. In one study, 68% of students in the sample changed their answer on at least one item, and out of that 68%, 45% of the examinees increased their scores, leading to an overall improvement [24]. Furthermore, the researchers argued that more proficient examinees are more likely to review more items and are more likely to change a wrong answer to the right answer [24]. Students spending more time per question often had higher Step 2 CK scores [9].

Extracurricular activities such as volunteering, tutoring, and club leadership are often completed by medical school students to appear more competitive for residency applications. Interestingly, volunteering, such as that at a student-run free clinic, had shown to have a positive correlation to Step 2 CK scoring [6,25]. On the other hand, leadership training courses did not seem to have a significant impact on Step 2 CK scoring [26].

With respect to​​*​​​​​ *psychological variables, self-reported stress during the third year was not correlated to Step 2 CK score [11]. There was a negative relationship between self-designated disadvantage (SDA) and Step 2 CK [3]. SDA is classified by the AAMC through a yes/no answer on the American Medical College Application Service (AMCAS) application to the question: “Do you wish to be considered a disadvantaged applicant by any of your designated medical schools that may consider such factors (social, economic or educational)?” [27]. While the AMCAS does not define disadvantage, previous studies consider it a “proxy for psychological drivers of academic performance” [3].

A longer time spent in medical school, defined by more than four years, but not including additional programs, was seen to have a negative correlation to Step 2 CK scoring [28]. In addition, recent matriculants from the years 1978-1991 were more likely to pass NBME parts 1 and 2 (which later became Step 2 CK of the USMLE sequence) [29].

Lag time was defined by Jurich et al. in a 2020 study as the time between the end of core clerkships and the first Step 2 CK attempt. Their results showed that Step 2 CK scores declined with an increasing lag time [8]. Students with longer lag time often took Step 1 and Step 2 CK after clerkships. Students taking Step 1 after clerkships and therefore delaying their Step 2 date (lag time: ~200 days) performed worse on Step 2 CK compared to students that took Step 1 before clerkships (lag time: ~100 days) [8].

Specific study tools, such as using mechanistic case diagrams, a form of concept mapping, can help with the integration of knowledge connected to clinical reasoning. For some students, this technique may also help increase Step 2 CK scores [30].

Institutional variables: These variables comprise factors that the institutional can change, such as preclinical characteristics, clerkship characteristics, faculty characteristics, and institutional characteristics.

Preclinical characteristics are defined as attributes of the academic curriculum for an institution, such as courses and grading prior to clinical training. Over the last decade, many medical school administrations have decided to adopt a pass/fail grading scheme as opposed to a tiered categorical grading system. Some students may worry that admission to a medical institution with the tiered categorical grading scheme will be detrimental. We found that a pass/fail grading curriculum had no significant impact on Step 2 CK scoring [4,5]. However, individual preclinical examination grades did demonstrate a small effect size on Step 2 CK scoring [16].

It seems that school-specific curriculum choices also did not have a significant impact on Step 2 CK scoring. Take the example of an important first-year course - anatomy. The manner of anatomical instruction, stand-alone versus integrated and dissection versus dissection/prosection, did not have a significant impact on Step 2 CK scoring [31].

Studies have shown that there is not a significant relationship between the clerkship sequence and Step 2 CK scoring [32,33]. One study found that IM clerkship characteristics and community-based medicine were not significantly associated with mean Step 2 CK scores [34]. However, variables such as seeing more patients in a day during the third year were positively correlated to Step 2 CK scoring (R^2^ = 0.47, p < 0.01) [34]. One study also found that students completing IM and then surgery clerkship had higher surgery subject examination scores and that a one-point increase in the surgery NBME subject examination score increased the odds of passing Step 2 CK by 1.2 times [21].

A reintroduction of basic science fundamentals also seemed to have a positive effect on Step 2 CK scoring. Medical students participating in a third-year basic science course at the University of South Carolina School of Medicine scored four points higher on Step 2 CK than their classmates that did not take the course [35].

The faculty characteristics that held relevance to Step 2 CK scoring were preclinical faculty-to-student ratio and National Institute of Health (NIH) funding statistics. Full-time faculty-to-student ratios (r = 0.35, p < 0.0004), total NIH (r = 0.46, p < 0.0001), and per faculty NIH funding (r = 0.35, p < 0.0005) were positively correlated with Step 2 CK score [36].

Variables within the category institutional characteristics include interview style, curriculum and educational policies, private versus public institution, and availability of a BA/MD program.

Studies found that private medical school students have a higher average Step 2 CK score compared to students at public medical schools [36,37]. Another study found no significant relationship between an institution’s educational policy and curriculum with Step 2 CK score [38]. Participation in a BA/MD accelerated program was also not significantly associated with Step 2 CK scoring [39].

Institutions often use interviews to determine best-fit students for their prospective medical school class. These interviews may also shed light on future USMLE performance. A study found that Multiple Mini Interview (MMI) scores were associated with higher mean Step 2 CK scores. They found that a single standard deviation increase in MMI score led to a 1.25-point increase in Step 2 CK [40]. No such relationship was found for traditional interviews.

Unmodifiable Factors

Unmodifiable factors are factors determined prior to enrollment into medical school and cannot be modified before the first Step 2 CK attempt.

Medical College Admission Test (MCAT): Many studies have shown that the MCAT score was a strong predictor for Step 2 CK score [36,41]. Two studies (p = 0.04 and p < 0.001) showed that a positive relationship exists when using the older style of MCAT scoring in regard to Step 2 CK performance [42]. Studies have shown that the specific individual sections - biological sciences (BS), physical sciences (PS), and verbal reasoning (VR) - of the MCAT have relevance to the Step 2 CK performance. One study found that the strongest MCAT section predictor was the BS section (r = 0.18, p = 0.001) [10], while another noted that there was a positive and significantly correlated relationship to the MCAT VR section (p = 0.037) [12]. Yet another study found that all three sections of the MCAT positively correlated with Step 2 CK performance (p < 0.001) [43]. Specifically, Step 2 CK score increased 2.819 points for a one-point increase on BS, 0.822-point increase on PS, and 1.238-point increase on VR [43].

Furthermore, there is a negative correlation between MCAT attempts and Step 2 CK performance, such that having more attempts led to lower performance on the Step 2 CK examination (r = -0.182, p = 0.000) [23]. There also seems to be a negative correlation between the time taken during the MCAT and Step 2 CK performance given that students using extra time on the MCAT examination had lower pass rates for Step 2 CK (p < 0.001) [44].

Undergraduate GPA: Both undergraduate total GPA [36] and science GPA [10] were shown to have a positive correlation to Step 2 CK score and pass rate. When comparing both metrics, science GPA was shown to have a more significant correlation to Step 2 CK score than total GPA [10].

Demographics: Variables within this category include age, race/underrepresented minority (URM), gender, socioeconomic disadvantage (SED), and language/English as a second language (ESL).

Many studies found that older students often performed worse on Step 2 CK [10,45]. One study from The University of Toledo College of Medicine (cohort 1998-2004) found that medical students younger than 22 had an average Step 2 CK score of 220, those between 23 and 25 had an average score of 214.7, and those older than 26 had an average score of 206.5 [10]. It should be noted that the passing score for Step 2 CK was 170 in 1998 and 182 in 2004 [46].

There also seems to be a significant correlation between gender and Step 2 CK performance [17,45]. Studies have shown that women outperform men on Step 2 CK and are also more likely to pass on the initial attempt. One study developed a model that predicted women to have a score 0.34 points higher than men [45], and a study at the Meharry Medical College found that male students scored eight points less than female students [12]. Interestingly enough, studies have shown that men outperform women on Step 1 [9,38].

There also exists a relationship between race, particularly URM, and Step 2 CK scoring. One study mentioned that there is a significant association specifically with African Americans and Step 2 CK but did not explore the nature of that association [35]. However, another study involving 818 students at The University of Toledo College of Medicine did find a negative correlation, showing that African Americans tend to score lower on the Step 2 CK examination compared to their peers (p = 0.001) [10]. Some research, however, does seem to question the relationship, if any, between race and Step 2 CK performance [23]. Speaking English as the primary language seems to have a positive impact on Step 2 CK performance [9], and test-takers who have English as their secondary language often scored lower on average than their peers [45].

## Conclusions

Our findings suggest that academic success starting from the undergraduate level and continuing on to medical school has a positive influence on eventual Step 2 CK scoring. Particularly important factors included the Step 1 score, USMLE practice examinations (CCSSA and CCSE), and clinical evaluations (NBME, clinical block grades, etc.). Students can also modify their behaviors during the examination; this may improve performance by increasing the time used per question and reducing the fear associated with changing answers. Interestingly, institutional characteristics such as a pass/fail versus traditional preclinical grading system did not influence Step 2 CK scoring. This should ease medical students’ concerns about their program’s specific grading attributes. Table 1 provides a more detailed analysis of the articles used for this paper’s synthesis.

This review has multiple limitations. First, many of the included factors were evaluated in a few studies, and data were not conclusive. Such factors include campus assignment, overall clerkship sequence, URM status, private versus public medical institution, lag time, stress, and leadership qualities. Some of the above variables showed statistical insignificance, while others were only discussed infrequently. Furthermore, in some of the studies, the confounding variables were not well controlled for by the study investigators. Due to the historically significant emphasis placed on Step 1, there have been many studies looking into specific study tools to increase examination performance. On the other hand, we did not find current research evaluating specific tools that positively correlate with the Step 2 CK score. Further research is needed to maximize performance on this examination and increase the chances for medical institutions to have more successful match outcomes.

## Appendices

**Table 1:** Summary of PRISMA studies SED: socioeconomic disadvantage; SDA: subjective self-designated disadvantage; GPA: undergraduate grade point average; USMLE: United States Medical Licensing Examination; NBME: National Board of Medical Examiners; CBSE: Comprehensive Basic Science Examination; CCSE: Comprehensive Clinical Science Examination; CCSSA: Comprehensive Clinical Science Self-Assessment; OSCE: Objective Structured Clinical Examination; NIH: National Institute of Health; ESL: English as a second language; MCAT: Medical College Admissions Test; BS: biological sciences section of MCAT; PS: physical sciences section of MCAT; VR: verbal reasoning section of MCAT; MMI: Multiple Mini Interview; FSMB: Federation of State Medical Board; SRS: Student Record System; MSQ: Matriculating Student Questionnaire; MCD: mechanistic case diagraming; SI: Supplemental Instructor; United States medical graduates: USMGs

Study	Sample size, study design	Variables	Findings
Jerant et al., 2019 [3]	N = 531	SED/SDA	Unadjusted SED+/SDA+ had the lowest mean Step 2 CK scores. Adjusted SED+/SDA+ and SED-/SDA+ students had lower scores on Step 2 CK. However, SED was not specifically associated with Step 2 CK performance.
Kim et al., 2018 [4]	N = 96 medical schools	Pass/fail curriculum	MCAT was a strong predictor of Step 2 CK (p < 0.001, b = 1.13 (0.13), r^2^ = 0.45). Undergraduate GPA was not a significant predictor of Step 2 CK (p = 0.55). After adjusting for MCAT, pass/fail grading was not significantly associated with Step 2 CK (p = 0.63).
Bloodgood et al., 2009 [5]	N = 281	Curriculum changes	Noninferior effect on Step 2 CK. A two-tailed t-test showed no statistically significant difference (p = 0.060) between the pass/fail and graded classes.
Vaikunth et al., 2014 [6]	N = 689, observational	Service at a student-run clinic	Volunteers (240 (18)) had higher Step 2 CK scores compared to peers that were non-volunteers (230 (21)) (p < 0.001).
Wong et al., 2007 [7]	N = 199	Peer-led teaching during medical school	Step 2 CK scores for SI leaders and non-SI leaders were significantly different (p < 0.001) (SI leader Step 2 CK = 214.4 and non-SI leader Step 2 CK = 221.7); matching for the year of enrollment, age, gender, MCAT score, and admission GPA).
Jurich et al., 2020 [8]	N = 3,199, retrospective	Step 1 examination timing, lag time, and MCAT score	Step 2 CK performance did not change significantly after Step 1 timing change (p = 0.2). Failure rates on Step 2 CK also remained constant (1.83% before and 1.79% after). Lag time had a significant negative effect on Step 2 CK performance (p < 0.001). Small, significant interaction effects between MCAT and Step 2 CK score (p = 0.005).
Cuddy et al., 2006 [9]	N = 54,487, observational	Step 1 score, gender, time per Step 2 CK question, percent of female students, percent of native English speakers, and average Step 1 score	Higher scores as regards to English as primary language (219.08 (22.30)), women (219.83 (21.59)), and more time per question (218.84 (22.83)). Step 1 score was positively correlated to Step 2 CK score (7.5-point increase in Step 2 CK for every 10-point increase in Step 1).
Kleshinski et al., 2009 [10]	N = 641, retrospective	Race, age, undergraduate major, total GPA, science GPA, and MCAT scores	African Americans had significantly lower mean Step 2 CK compared to other races (198.4 (18.3), p = 0.001). Age of matriculation was inversely related to Step 2 CK score (<22 = 220 (21.2); 23–25 = 214.7 (22.3); >26 = 206.5 (20.9)) and was statistically significant. No significant difference based on undergraduate major. Significant correlation of total GPA (p = 0.001), science GPA (p = 0.001), and BS section of MCAT to Step 2 CK score (p = 0.001).
Fetter et al., 2019 [11]	N = 70	Clinical NBME grades; stress	Respondents reported moderate levels of personal stress related to academic factors (2.0 (0.46)), and teaching and learning factors (1.9 (0.58)). Academic factors posing the severest stress were “doing well on Step 2 CK” (3.02 out of 4). Conversely, doing well on shelf examinations and “difficulty with clinical learning” posed mild stress (1.14–1.30). Neither subscale was associated with Step 2 CK (rSpearman = -0.09 and -0.02, respectively).
Chen et al., 2016 [12]	N = 196	NMBE CBSE, MCAT VR score, Step 1 score, and gender	Gender was a significant predictor for Step 2 CK (p < 0.001) as male students scored eight points less than female students. MCAT VR score was a positive significant contributor to USMLE Step 2 CK score (p < 0.05). Another significant contributor was the USMLE Step 1 score (p < 0.001) as a 10-point increase in the USMLE Step 1 contributed to a two-point increment in the USMLE Step 2 CK score. The highest explanatory variables correlated with the USMLE Step 2 CK were the NBME CBSE score from April examination (r = 0.89), the USMLE Step 1 (r = 0.88), and the NBME CBSE score (r = 0.77).
Morrison et al., 2014 [13]	N = 4,722, retrospective	NBME Comprehensive Clinical Science Self-Assessment (CCSSA) scores	CCSSA examination scores explained 58% of the variation in the first attempt Step 2 CK scores for USMGs and were also significant predictors (p < 0.01). Students with low CCSSA scores were at risk for failing Step 2 CK.
Morrison et al., 2018 [14]	N = 3,736, retrospective	CCSE examination scores	CCSE was a significant predictor for Step 2 CK score in USMGs (r^2^ = 0.48, p < 0.01). Regression models explained 50% of the total variance between CCSE and Step 2 CK scores. Higher CCSE is associated with a greater probability of passing Step 2 CK (OR = 1.191). Nearly perfect probability of passing Step 2 CK with a CCSE score of 90 or above.
Guiot et al., 2018 [15]	N = 564	CCSE examination scores	A significant correlation (r = 0.572, p ≤ 0.001) was found between the score in the NBME Medicine CCSE and the score in the USMLE Step 2 CK. There was a significant correlation (r = 0.698, p ≤ 0.001) between the scores in the USMLE Step 1 and the USMLE Step 2 CK. There was a significant correlation (r = 0.684, p ≤ 0.0001) between obtaining a score of 208 or higher in the USMLE Step 1 and subsequently attaining a passing grade on the first take of the USMLE Step 2 CK.
Monteiro et al., 2017 [16]	N = 218	Clinical NBME grades, Step 1 score, and preclinical grading	Mean preclinical course examination score demonstrating a small effect size (B = 0.17, t = 3.11, p = 0.002) and Step 1 score demonstrating a large effect size (B = 0.64, t = 11.52, p < 0.001). Both Step 1 score (B = 0.27, t = 4.27, p < 0.001) and subject examinations (B = 0.54, t = 8.46, p < 0.001) were significant predictors of Step 2 CK score.
Cuddy et al., 2007 [17]	N = 23,538, retrospective	Gender and Step 1 score	Women outperformed men in most Step 2 CK content areas. Specifically, in OB/GYN (observed difference = 7.6), gynecologic disorders (observed difference = 7), and disorders of pregnancy (observed difference = 5.7) with p < 0.01. Step 1 scores and gender were significant predictors within schools. Step 1 scores were positively related to Step 2 CK content area scores. Step 1 scores for men were more associated with Step 2 CK scores than for women.
Durning et al., 2015 [18]	N = 1,255	Age, clinical block grades, preclinical GPA, Step 1 score, undergraduate science GPA, and total GPA	Significant small correlations were found between board certification and IM clerkship points (r = 0.117), IM clerkship grade (r = 0.108), clerkship year GPA (r = 0.078), undergraduate college science GPA (r = 0.072), pre-clerkship GPA and medical school GPA (r = 0.068 for both), USMLE Step 1 (r = 0.066), undergraduate college total GPA (r = 0.062), and age at matriculation (r = -0.061).
Dong et al., 2012 [19]	N = 802	OSCE scores	Second-year OSCE score had weak correlations with Step 2 CK score (r = 0.14, p < 0.01). Third-year OSCE score had weak correlations with Step 2 CK score (r = 0.14, p < 0.01). Additional USMLE Step 2 CK score variance accounted by the second- and third-year OSCE scores beyond that explained by the Step 1 score was minimal (r^2^ change = 0.01).
Simon et al., 2007 [20]	N = 340	OSCE scores and Step 1 score	Total OSCE score correlation to Step 2 CK was moderate (r = 0.395, p < 0.001). Step 1 and 2 CK highly correlated (r = 0.723, p < 0.001). Five of the seven OSCE skills subcomponents were significantly correlated with Step 2 CK scores. Most significant were differential diagnosis (r = 0.343, p < 0.001) and identification of abnormalities (r = 0.322, p < 0.001). Step 1 score accounted for 57.5% of variability. Addition of OSCE only accounted for small increase (1.3%) in Step 2 CK variability.
Dong et al., 2018 [21]	N = 687, retrospective	Clerkship sequence and MCAT score	Students completing IM and then surgery clerkship had higher surgery subject examination scores. A one-point increase in NBME surgery score increased the odds of passing Step 2 CK by 1.2 times. The odds of passing Step 2 CK for students completing surgery and then IM were seven times higher than for students completing IM and then surgery. Surgical NBME and surgical clerkship final score had moderate correlation to Step 2 CK score (r = 0.56, p < 0.01 and p < 0.01, respectively).
Zahn et al., 2012 [22]	N = 484 out of 507 had complete data sets	Clinical NBME grades	Correlation between average subject examination scores across all six clerkships and the Step 2 CK examinations were quite strong (r = 0.77, p < 0.001). USMLE Step 2 CK scores were also positively correlated with all explanatory variables, with correlations ranging from 0.51 (95% CI: 0.44–0.57, p < 0.01) to 0.68 (95% CI: 0.63–0.73) (p < 0.01).
Ogunyemi and Taylor-Harris, 2005 [23]	N = 171	Age, race, gender, MCAT attempts, clinical NBME grades, and undergraduate GPA	Significant correlation between Step 2 CK score and undergraduate GPA (r = 0.287), MCAT score (r = 0.524), Step 1 score (r = 0.681), and NBME OB/GYN score (r = 0.614). No correlation with race or gender. Negative correlation between Step 2 CK score and increasing age (r = -0.405), increasing MCAT attempts (r = -0.182), and increasing NBME OB/GYN attempts (r = -0.310). Variables associated with failing Step 2 CK score were failing NBME OB/GYN score (p = 0.008), Step 1 failing score (p = 0.01), and multiple attempts on MCAT (p = 0.033).
Ouyang et al., 2019 [24]	N = 27,830	Changing answers on Step 2 CK	The average increase in CK scores is associated with changing answers based on examination proficiency. Of the examinees, 68% changed at least one item, and among this group, 45% increased their scores and 28% decreased their scores.
Blue et al., 2006 [25]	N = 263	Volunteerism	Students in the highest service group (>18.5 hours) had significantly higher Step 2 CK scores before and after controlling for premedical GPA (p = 0.0086).
Barry et al., 2019 [26]	N = 483 out 509 (complete data sets available)	Leadership qualities	Analyses revealed that leader performance was not correlated with students’ performance on Step 2 Clinical Knowledge examination score (r = 0.09, p = 0.06).
Arvidson et al., 2015 [28]	N = 1,328	Extended curriculum time	Students taking extended curriculum time had first-time Step 2 CK pass rate of 83% compared to 97% of their peers (χ² = 53.24, p < 0.001).
Andriole et al., 2012 [29]	N = 6,594, observational, cohort 1978–1991	AAMC SRS and MSQ answers, MCAT score, and Step 1 pass/fail status and score	More likely to initially pass Step 2 CK: women, higher MCAT scores, and recent matriculants. Less likely to initially pass Step 2 CK: Asian/Pacific Islanders or URM, older, Step 1 scores in the lowest or middle tertiles (97–173), and attended private medical schools. All significant relationships had a p < 0.05 and 95% confidence intervals.
Ferguson et al., 2020 [30]	N = 136	Concept mapping and case diagrams	Students’ overall MCD scores correlated significantly with standardized examination measures USMLE Step 2 Clinical Knowledge (r = 0.39, p < 0.0001).
Cuddy et al., 2013 [31]	N = 5,782, observational	Anatomical course instruction (integrated versus stand-alone)	Anatomical instruction did not have practical importance on Step 2 CK total score due to the small effect size.
Kies et al., 2010 [32]	N = 2,236, retrospective	Gender, campus, and Step 1 score	Step 2 CK was associated with sex, campus, and Step 1 score (p < 0.001). Women had higher Step 2 CK scores than men. Step 1 had the strongest contribution to Step 2 CK score.
Gao et al., 2019 [33]	N = 135	Clerkship sequence	Wilks’s statistic found no statistically significant effect of rotation sequence (starting the clerkship year in FM or IM) on the pediatrics, surgery, and Step 2 CK examinations (Λ = 0.95, F (3,51) = 0.93, p ≤ 0.432). Wilk’s statistic found no statistically significant effect of rotation sequence (starting the clerkship year in pediatrics or surgery) on the IM and Step 2 CK results (Λ = 0.925, F (2,75) = 3.036, p ≤ 0.054).
Griffith III et al., 2009 [34]	N = 1,817	Clerkship characteristics, small group hours per week, number of patients seen per day during IM rotation, and community-based medicine	No variable as regards to IM clerkship characteristics (length of clerkship, small group hours per week, average number of patients cared for by student per day, and community-based medicine versus not) was found to be significantly associated with mean Step 2 CK scores (school level). Percentage of students with significant improvement (at least 0.5 SD or 10 points or more) from Step 1 to Step 2 CK was associated with a greater average number of patients cared for by students per day (R^2^ = 0.47, p < 0.01). The inverse was also significantly associated (R^2^ = 0.44, p < 0.02), i.e., fewer patients cared for less to a greater percentage of students with score drops from Step 1 to Step 2 CK.
Brownfield et al., 2008 [35]	N = 743	Foundations of Clinical Medicine (FCM) small group course	Post-FCM cohort mean unadjusted Step 2 CK score (215.9 +/- 21.9) significantly higher than pre-FCM cohort (207.7 +/- 22.1) with p < 0.001. Post-FCM students scored four points higher on Step 2 CK after adjusting for the variables mentioned in the findings. Overall, the variables FCM cohort (p = 0.0005), Step 1 score (p < 0.0001), African American race (p < 0.008), age (p < 0.0001), and gender (p < 0.0001) were significantly associated with Step 2 CK score.
Ghaffari-Rafi et al., 2019 [36]	N = 100 medical schools	Step 1, median GPA, median MCAT, full-time faculty-to-student ratio, NIH funding, and public versus private schools	Statistically significant correlations with Step 2 CK and Step 1 score (r = 0.54, p < 0.0001), median GPA (r = 0.49, p < 0.0001), median MCAT total score (r = 0.60, p < 0.0001), full-time faculty-to-student ratio (r = 0.35, p = 0.0004), NIH funds granted to medical schools and affiliated hospitals (r = 0.46, p < 0.0001), and NIH research grant funds per faculty member (r = 0.35, p = 0.0005). Compared to public schools, private schools have a slightly higher Step 2 score (241.3 versus 239.2, p = 0.051).
Burk-Rafel et al., 2019 [37]	N = ~390,000	Undergraduate GPA, MCAT, and demographic variables	Step 2 CK was significantly related to institutional GPA and MCAT, Step 1, minority students and biological science majors and institutions with NIH funding (not significant after controlling for MCAT and GPA), and private institutions (either p < 0.05 or 0.01).
Poon et all., 2019 [38]	N = 9,133	Gender and Step 1 score	Step 1 scores were higher in men than in women (p < 0.0001).
Green et al., 2016 [39]	N = 2,583	BA/MD program	Students in Honors Program in Medical Education (HPME) (236.8 (19.6)) did not have significantly different Step 2 CK scores compared to non-HPME peers (237.7 (19.9)) with p = 0.41.
Jerant et al., 2019 [40]	N = 1,460, observational	MMI versus traditional medical school interview	MMI association with Step 2 CK was significant (p = 0.04), while traditional was not (p = 0.49).
Shah et al., 2018 [41]	N = 227, observational	Preclinical volunteer experience, undergraduate GPA, and MCAT	Preclinical volunteer experience (p = 0.01), undergraduate GPA (p = 0.01), and MCAT scores (p < 0.01) positively predicted Step 2 CK score.
Bills et al., 2016 [42]	N = 153, retrospective	MCAT score and undergraduate GPA	Cohort II MCAT scores (1978–1991) were associated with Step 2 CK score (p = 0.04) but overall were inconsistent predictors.
Gauer et al., 2016 [43]	N = 1,065, retrospective	MCAT score	Significant moderately positive relationship between MCAT composite and Step 2 CK (r = 0.31, p < 0.001). BS, PS, and VR sections of MCAT were significant predictors of Step 2 CK (p < 0.001, p = 0.007, p < 0.001) and accounted for 12% of Step 2 CK variance. Step 2 CK score increased 2.819 points for a one-point increase on BS, 0.822-point increase on PS, and 1.238-point increase on VR.
Searcy et al., 2015 [44]	N = 211,108	Standard versus extra administration time during MCAT	Lower Step 2 CK pass rate for students using extra time (difference = 9.9%) (χ² = 89.34, p < 0.001).
Rubright et al., 2019 [45]	N = 45,154	Age, gender, and English as the primary language	Of the variance in Step 2 CK scores, 90% was due to student differences. All demographic variables under study were statistically significant.
